# Impact of Glycerol as Carbon Source onto Specific Sugar and Inducer Uptake Rates and Inclusion Body Productivity in *E. coli BL21*(*DE3*)

**DOI:** 10.3390/bioengineering5010001

**Published:** 2017-12-21

**Authors:** Julian Kopp, Christoph Slouka, Sophia Ulonska, Julian Kager, Jens Fricke, Oliver Spadiut, Christoph Herwig

**Affiliations:** 1 Christian Doppler Laboratory for Mechanistic and Physiological Methods for Improved Bioprocesses, Institute of Chemical, Environmental and Biological Engineering, Vienna University of Technology, 1060 Vienna, Austria; julian.kopp@tuwien.ac.at (J.K.); christoph.slouka@tuwien.ac.at (C.S.); fricke_jens@gmx.de (J.F.); 2 Research Division Biochemical Engineering, Institute of Chemical, Environmental and Biological Engineering, Vienna University of Technology, 1060 Vienna, Austria; sophia.ulonska@tuwien.ac.at (S.U.); julian.kager@tuwien.ac.at (J.K.); oliver.spadiut@tuwien.ac.at (O.S.)

**Keywords:** *E. coli*, mixed feed system, glycerol, recombinant proteins, bioprocess engineering

## Abstract

The Gram-negative bacterium *E. coli* is the host of choice for a multitude of used recombinant proteins. Generally, cultivation is easy, media are cheap, and a high product titer can be obtained. However, harsh induction procedures using isopropyl β-d-1 thiogalactopyranoside as inducer are often referred to cause stress reactions, leading to a phenomenon known as “metabolic” or “product burden”. These high expressions of recombinant proteins mainly result in decreased growth rates and cell lysis at elevated induction times. Therefore, approaches tend to use “soft” or “tunable” induction with lactose and reduce the stress level of the production host. The usage of glucose as energy source in combination with lactose as induction reagent causes catabolite repression effects on lactose uptake kinetics and as a consequence reduced product titer. Glycerol—as an alternative carbon source—is already known to have positive impact on product formation when coupled with glucose and lactose in auto-induction systems, and has been referred to show no signs of repression when cultivated with lactose concomitantly. In recent research activities, the impact of different products on the lactose uptake using glucose as carbon source was highlighted, and a mechanistic model for glucose-lactose induction systems showed correlations between specific substrate uptake rate for glucose or glycerol (q_s,C_) and the maximum specific lactose uptake rate (q_s,lac,max_). In this study, we investigated the mechanistic of glycerol uptake when using the inducer lactose. We were able to show that a product-producing strain has significantly higher inducer uptake rates when being compared to a non-producer strain. Additionally, it was shown that glycerol has beneficial effects on viability of cells and on productivity of the recombinant protein compared to glucose.

## 1. Introduction

The Gram-negative bacterium *E. coli* is the expression host of choice for the production of 30% to 40% of recombinant drugs in industry [1,2]. As *E. coli* shows very fast replication rates [3,4] on comparatively cheap media [5], the benefits often outweigh the numerous purification steps [1,6] and the missing glycosylation pattern [1,7,8]. Recombinant protein production in *E. coli* gained more interest again as the demand in single-chain antibody fragments increased, which can be properly expressed in *E. coli* [1,8]. The strain *BL21*(*DE3*), created by F. Studier and B. Moffatt back in 1986 [9], is often used in an industrial scale because of very low acetate formation, high replication rates as an effect of the integrated T7-polymerase [9,10,11,12,13,14], as well as the possibility of protein secretion into the fermentation broth due to a type 2 secretion protein [15,16,17]. As the lac operon is still one of the most favored promotors in pET-expression systems [3,12,18], it is generally used for insertion of the gene of interest. The repressor protein can only be blocked by allolactose or a structural analogue [19], e.g., the well-known inducer isopropyl β-d-1 thiogalactopyranoside (IPTG) [3,13]. However, induction with IPTG stresses the cells, as IPTG in higher concentrations is referred to be toxic at elevated induction times [13,18,20]. As tunable protein production is commonly applied in industry nowadays, mixed-feed systems using either IPTG [21] or lactose [13,22,23] as inducer did result in higher product yields when compared to other inducer supplies [24]. Soft induction performed with lactose shows promising results [13,23,25]. As lactose can be metabolized in *E. coli*, it does not stress the cells as much as IPTG [26]. For the production of soluble proteins, induction with lactose usually is preferred [3], but it has also been shown that lactose shows promising results for Inclusion Bodies (IBs) and products located in the periplasm [3,27]. 

IBs have originally been believed to be waste products by bacteria [28], until it was realized that IBs tend to form as a stress reaction by the cells resulting in a biologically inactive protein [29,30,31]. Stress reactions of the cells can be caused by high temperatures, pH-shifts, or due to high feeding rates. Higher feeding rates result in higher yields of product [1], which of course is advantageous when combined with the possibility of expressing toxic proteins [6]. Still, the downstream process (DSP), and especially the refolding unit operation, is the time-consuming step in gaining the correctly folded product from *E. coli* cultivations [28,29,30,31], which requires significantly more technology and time in purifying IBs [29,32,33]. Though IBs can be produced in such excess, the amount of generated product often outweighs the DSP efforts and makes the time-space yield more preferable for IBs [1,6,7,28].

One of the most favoured carbon sources in *E. coli* cultivations has always been glucose, as glucose has a very high affinity to the phosphotransferase system [34,35]. Glucose provides a lot of energy for the cells, as it is directly induced into glycolysis as glucose 6-phosphate and consumed through the tricarboxylic acid cycle (TCA) [35,36]. Usage of such, in combination with lactose, may result in diauxic growth and catabolite repression, which are caused by the regulatory network that is induced by glucose [37,38,39]. Catabolite repression results in decreased lactose uptake rates when glucose is present in excess [27,39,40]. Glycerol, first noticed in biotechnology as a by-product in the biodiesel production [41], has shown quite promising results in terms of biomass/substrate yield in *E. coli* cultivations [22,25]. To our knowledge, up to this point, no catabolic repression has been reported when glycerol was used as main carbon source (C-source) in combination with lactose [42]. In addition, mixtures of glucose, glycerol, and lactose have shown promising results for diverse products gained via autoinduction systems [20,25]. Recent studies [3,40] showed that the dependence of the inducer lactose influences the maximum IB production even on a quite low level of the specific glucose uptake rate. Low feeding rates of glucose would therefore result in the maximum inducer uptake rate, as cyclic adenosine monophosphate (cAMP) levels increase at higher glucose addition and therefore decrease the affinity for the RNA polymerase, decreasing the expression of the genes coding for the lac operon [35]. It is believed that cultivations with glycerol are able to overcome the problem of carbon catabolite repression and pave the way for usage of much higher specific C-source uptake rates, in order to increase time-space yields.

In this study, we performed cultivations with a *BL21*(*DE3*) strain, producing a recombinant protein coupled to a N-pro-fusion protein [43], expressed as IB with the goal to yield in maximum recombinant protein production. It is believed that glycerol causes positive results for the mixed-feed optimization when using lactose as an inducer, as glycerol—introduced into glycolysis but also into gluconeogenesis—yields a high amount of energy supplied to the cultivation system [42,44,45]. Couple that with increased cAMP levels throughout the whole cultivation, [35] glycerol is believed to be beneficial over a glucose cultivation system. It is shown that the recombinant protein production is increased compared to glucose, as a result of more available energy.

## 2. Materials and Methods 

### 2.1. Bioreactor Cultivations

All cultivations were carried out with the strain *E. coli BL21*(*DE3*) consisting of the pET-30a plasmid system. The eukaryotic target protein was linked to a N-pro fusion taq (size of 28.8 kDA for the fusion protein) [43]. As the given protein is currently under patenting procedure at the industrial partner no detailed information can be given on the used protein.

All bioreactor and preculture cultivations were carried out using a defined minimal medium referred to DeLisa et al. (2015) [5]. Batch media and the preculture media had the same composition with different amounts of sugars respectively. The sugar concentrations for the phases are presented in Table 1:

As pET-30a has a Kanamycin resistance gene, antibiotic was added throughout all fermentations, resulting in a final concentration of 0.02 g/L. All precultures were performed using 500 mL high yield flasks (containing the sugar concentrations given in Table 1). They were inoculated with 1.5 mL of bacteria solution stored in cryos at −80 °C and subsequently cultivated for 20 h at 230 rpm in an Infors HR Multitron shaker (Infors, Bottmingen, Switzerland) at 37 °C.

All cultivations were either performed in a DASGIP Mini bioreactor-4-parallel fermenter system (max. working volume: 2.5 L; Eppendorf, Hamburg, Germany) or in a DASbox Mini Bioreactor 4-parallel fermenter system (max. working V.: 250 mL; Eppendorf, Hamburg, Germany). For measuring the CO_2_ and O_2_ flows, a DASGIP-GA gas analyser was used (Eppendorf, Hamburg, Germany). The cultivations were controlled using the provided DAS-GIP-control system, DASware-control, which logged the process parameters. During cultivation, pH was kept constant at 7.2 and controlled with base only (12.5% NH_4_OH), while acid (10% H_3_PO_4_) was added manually, if necessary. The pH was monitored using a pH-sensor EasyFerm Plus (Hamilton, Reno, NV, USA). Base addition was monitored observing the flowrates of a DASbox MP8 Multipumpmodul. The reactors were continuously stirred at 1400 rpm.

Aeration was absolved using mixture of pressurized air and pure oxygen at 2 vvm, mixing the ratios of the airflow, so that the dissolved oxygen (dO_2_) was always higher than 40%. The dissolved oxygen was monitored using a fluorescence dissolved oxygen electrode Visiferm DO 120 (Hamilton, Reno, NV, USA).

### 2.2. Cultivation Scheme and q_s_ Screening Procedure

The batch media in the DASGIP reactors always contained 1 L DeLisa medium, while the DASbox Mini bioreactors contained a volume of 100 mL.

Only static q_s_-controls were performed for these experiments, as the q_s,C_ was not altered during induction phase [3,27]. The procedure was always as follows: Preculture, Batch, non-induced fed-batch, and induced fed batch with an adapted q_s,C_.

Inoculation was always done with one tenth of the batch media volume, resulting in 100 mL of preculture. Preculture showed an OD_600_ of approximately 7 after cultivation (described above). The batch process, performed at 37 °C, took around 6–7 h, depending on the C-source used, and was finished, visible by a drop in the CO_2_ signal. The 22 g/L of either glucose or glycerol usually resulted in a biomass of 9–10 g/L. After the batch was finished, a non-induced fed-batch was started overnight, at 35 °C and adapting the q_s,C_ value to gain a biomass of approximately 30 g/L. After the non-induced fed-batch, the volume was always decreased to 1 L, in order to keep induction conditions the same. Afterwards, q_s,C_ was adapted to a certain point of interest, and temperature was decreased to 30 °C and stabilized for 30 min before the inducer was added. Induction was always performed with a lactose pulse of 100 mL of a 300 g/L sterile lactose solution—resulting in a lactose concentration in the fermentation broth of approximately 30 g/L. Induction period always lasted 7 h. The q_s_ control used here was performed using Equation (1) according to an exponential feed forward approach to keep q_s_ constant [3,27,40,46]:

(1) $$F\left(t\right)=\frac{q_{s,\;C}\times X\left(t\right)\times \rho_{f}}{c_{f}}$$

with F being the feed rate [g/h], q_s,C_ the specific glucose or glycerol uptake rate [g/g/h], X(t) the absolute biomass [g], ρ_f_ the feed density [g/L], and c_f_ the feed concentration [g/L], respectively.

### 2.3. Process Analytics

Samples are always taken after inoculation, upon end of the batch-phase and after the non-induced fed-batch was finished. During the induction period, samples were either taken in 20 or 30 min intervals. Generally, biomass was measured using OD_600_ and dry cell weight (DCW), while flow cytometry analysis (FCM) was used for determination of cell-death, especially in the induction phase. Optical density (OD_600_) was measured using a Genesys 20 photometer (Thermo Scientific, Waltham, MA, USA). Since the linear range of the used photometer is between 0.2 and 0.8 [AU], samples were diluted with dH_2_O to stay within that range. The dry cell weight was determined by vortexing the sample, pipetting 1 mL of sample solution in a pre-tared 2 mL Eppendorf-Safe-Lock Tube (Eppendorf, Hamburg, Germany), and centrifuged for 10 min at 11,000 rpm at 4 °C. After centrifugation, the supernatant was used immediately for at-line high-pressure liquid chromatography (HPLC) measurement (see beneath), while the pellet was re-suspended with 1 mL of 0.9% NaCl solution and centrifuged at the same conditions. Afterwards, the pellet was dried for at least 72 h at 105 °C. Samples for FCM were diluted 1:100 with 0.9% NaCl solution, stored at 4 °C, and measured after the process was finished. The measurement was performed using the software Cube 8 (Sysmex, Partec, Görlitz, Germany) according to Langemann et al. [47] using DiBAC_4_(3) (bis-(1,3-dibutylbarbituricacid) trimethineoxonol) and Rh414 dye. Rh414 binds to the plasma membrane and visualizes all cells, while DiBAC is sensitive to plasma membrane potential, and therefore distinction between viable and non-viable cells can be achieved.

Product samples were taken for [P]-strain, after 2, 5 and 7 h of induction phase. They were always treated as follows: 5 mL pipetted in a 50 mL Falcon tube, centrifuged for 10 min at 4800 rpm at 4 °C. The supernatant was discarded while the pellet was frozen at −20 °C. Samples for homogenisation were disrupted as follows: The pellets were re-suspended in a Lysis buffer (0.1 M TRIS, 10 mM EDTA, pH = 7.4) according to its dry cell weight (Equation (2)):

(2) $$\mathrm{Volume}\;\mathrm{Lysis}\;\mathrm{Puffer}=\mathrm{DCW}\times \frac{5}{4}$$

After suspending the cells, they were treated with an EmusiflexC3 Homogenizer (Avestin, Ottowa, ON, USA) at 1500 bar. The duration of homogenisation was always calculated to achieve ten passages through the homogenizer. After washing the pellets twice with dH_2_O, the samples were measured using a HPLC method. The N-pro-fusion protein IB was measured via RP-HPLC (Thermo Scientific, Waltham, MA, USA) using a Nucleosil-column after solving in 7.5 M Guanidine Hydrochloride based buffer. The eluent was a gradient mixture of water with 0.1% TFA (tri-fluoric-acid) and Acetonitrile mixed with 0.1% TFA with a flow of 3 mL/min. Standard concentrations were 50, 140, 225, 320 and 500 mg/mL of an industrial supplied reference.

Sugar and glycerol concentrations were measured via HPLC-method (Thermo Scientific, Waltham, MA, USA) using a Supelcogel-column; Eluent: 0.1% H_3_PO_4_; Flow: 0.5 mL/min. Using this method, glucose or glycerol accumulation as well as the lactose decrease and the galactose accumulation could be detected. Standards had a concentration of 0.5, 1, 5, 10 and 20 g/L of every sugar used throughout all fermentations. The HPLC run lasted always for 25 min and chromatograms were analyzed using a Chromeleon Software (Dionex, Sunnyvale, CA, USA).

## 3. Results and Discussion

### 3.1. Mechanistic Correlations of Glycerol onto Specific Lactose Uptake Rate

The basic feeding rate for the induction phase for production of the recombined protein is a constant q_s,C_—given by a fed-batch carried out on glucose or glycerol depending on the experiment—and by a pulse of 10 vol % high concentrated lactose feed. 

In order to get comparable datasets for all experiments, a mechanistic model approach is performed. As shown in previous studies, the maximum possible specific lactose uptake rates depend on the specific glucose uptake rates which can be described by a mechanistic equation (see Equation (3)) [3,40]. The maximum q_s,lac_ rates depend Monod-like on q_s,glu_ until a certain maximum is reached at a respectively low feeding rate of glucose, before q_s,lac_ decreases at high q_s,glu_ which performs analogue to substrate inhibition [3]. Values for y = 0 correspond to the uptake rates on sole glucose/glycerol, respectively.

(3) $$q_{s,\mathrm{lac}}=q_{s,\mathrm{lac},\max}\times \max\left({\left(1-\frac{q_{s,\mathrm{glu}}}{q_{s,\mathrm{glu},\mathrm{crit}}}\right)}^{n},\;0\right)\times \left(\frac{q_{s,\mathrm{glu}}}{q_{s,\mathrm{glu}}+K_{A}}+\frac{q_{s,\mathrm{lac},\mathrm{noglu}}}{q_{s,\mathrm{lac},\;\max\;}}\right)$$

with q_s,lac_ being the specific lactose uptake rate [g/g/h], q_s,lac,max_ the maximum specific lactose uptake rate [g/g/h], q_s,glu_ the specific glucose uptake rate [g/g/h], q_s,glu,crit_ the critical specific glucose uptake rate up to which lactose is consumed [g/g/h], q_s,lac,noglu_ the specific lactose uptake rate at q_s,glu_ = 0 [g/g/h], and K_A_ the affinity constant for the specific lactose uptake rate [g/g/h]. n describes the type of inhibition (non-competitive, uncompetitive, competitive).

**Figure 1:**
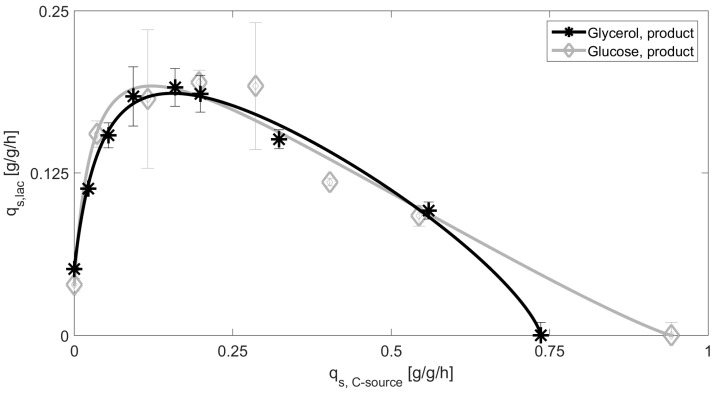
Extracted datapoints for q_s_ values including standard deviations for cultivations with glucose and glycerol in the production strain (glycerol product, glucose product). Solid lines represent the model based approach for inducer uptake rates vs. feeding rates models of glucose and glycerol.

As the model has already been established for four different products in glucose-lactose systems [40], it had to be shown if the same function fits for the given product. We fitted the model parameters as described in Wurm et al., where also a detailed description of the model derivation can be found [3]. As shown in Figure 1 and Table 2, parameters can be found to describe the experimental data for glucose and glycerol as C-source. In absence of glucose, lactose cannot be taken up, since there is not enough adenosine triphosphate (ATP) produced. Once a certain threshold of glucose is passed, enough ATP is created to metabolize the inducer [3,40]. The trend seen in the cultivations performed on glucose are explained by the well-known phenomenon of catabolite repression (CCR) [37,39], as the lactose uptake rates decrease significantly with increasing the feeding rate. As *E. coli BL21*(*DE3*) is not able to metabolize galactose due to absence of a (gal) gene, which can be referred to a deletion of the genes gal M, K, T, E [48,49], galactose should accumulate in the fermentation broth [37,50]. Hence, the galactose accumulation rate in the fermentation broth could generally be correlated to the lactose depletion rate during the cultivation (not shown).

However, the curves for glucose and glycerol are almost identical. Generally, a higher affinity for glucose is reported in literature [35], resulting in a higher µ for those cultivations, as glycerol has less affinity to the phosphotransferase system (PTS) [37]. This trend is in accordance with our data given in the value q_s,C,crit_ in Table 2. Furthermore, biomass to substrate yields (Y_X/S_) for glucose decrease in the induction phase from about 0.5 in the batch phase to about 0.336 ± 0.05 after the one-point lactose addition. By contrast, Y_X/S_ of glycerol are generally about 0.44 ± 0.1 during the induction phase [51].

This does not explain the very similar lactose uptake values at high q_s,C_, since it is believed that carbon catabolite repression should not be present using glycerol as primary carbon source [52]. The production of the recombinant protein seems to induce stress resulting in the maximum possible activity inside the cell, which is represented by the similarity of the two curves. Therefore, the decrease of the q_s,lac_ rate in the model-based approach actually referred to the CCR for glucose based systems so far (${\left(1-\frac{q_{s,\mathrm{glu}}}{q_{s,\mathrm{glu},\mathrm{crit}}}\right)}^{n})$, may have to be reconsidered when glycerol is fed. In turn, our results would indicate that the decline cannot be attributed to carbon catabolite repression, also not for glucose. Glycerol does not interfere with the PTS transport system and no resulting change of the cAMP levels during uptake of lactose are to be believed on a first glance. Glycerol enters glycolysis as di-hydroxy-acetone-phosphate and is processed in glycolysis producing pyruvate, but also there are gluconeogenetic genes active providing the formation of glucose-6-phosphate [41,53,54]. As glycolysis seems to be running at maximum capacity, a bottleneck in the trycarboxylic acid (TCA) cycle may also be likely. Overload of the TCA cycle has already been described by Heyland et al. (2011) [55], saying that the TCA cycle cannot metabolize all the pyruvate produced in glycolysis. It has also been referred that the cells try to gain energy in alternative ways such as using acetate as a terminal electron acceptor, or the usage of oxidative phosphorylation [55,56]. However, as *E. coli BL21*(*DE3*) produces relatively low levels of acetate in general, the acetate formation is always beneath the threshold of the HPLC and may therefore not the predominant electron acceptor in this strain.

**Figure 2:**
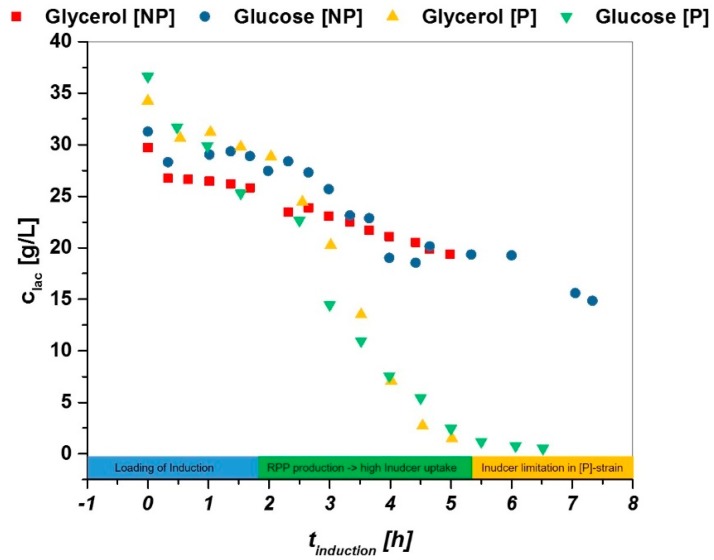
High-pressure liquid chromatography (HPLC)-based data for decrease of lactose in fermentation broth exhibiting very similar q_s,C_ values in [g/L]. A significant decrease over the time of induction is visible in producing (P) strains, while the decrease is way slower in non-producing (NP)-strain-cultivations.

To test the observed effects, we tried a process technological method approach, rather than performing expensive and time consuming “omics” analysis. The pET-30a plasmid was transformed into the used strain *E. coli BL21*(*DE3*) without the sequence for the recombinant protein, further referred as non-producer (NP) strain. The strain was tested in the same analytical way as the used strain for recombinant protein production. HPLC raw data for lactose decrease are compared with an almost identical q_s,C_ (~0,1 g/g/h) in Figure 2. 

Hereby, three phases can be seen for the product producing strain in the induction phase, while only two phases can be seen in the NP strain:

(i) Adaption phase: lactose gets transferred to alloactose and loads the induction (0–2 h in induction phase).
(ii) Linear decrease of lactose as the system needs inducer for recombinant protein expression (2–5 h).
(iii) Limitation of lactose in P strain: not sufficient inducer present, need for mixed feed system (5–7 h), no inducer limitation seen in NP strain, further decrease of inducer analogue to phase 2.

**Figure 3:**
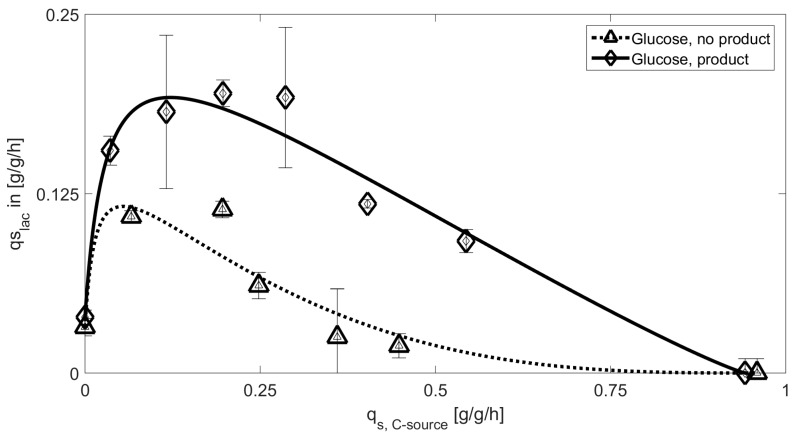
Extracted datapoints for q_s,C_ values including standard deviations for cultivations with glucose using the product producing (glucose product) and the NP strain (glucose, no product). Solid lines represent the model based approach for inducer uptake rates vs. feeding rates models of glucose. A clearly visible difference can be observed during these cultivations.

Results on the model-based approach for the glucose system are given in Figure 3.

**Figure 4:**
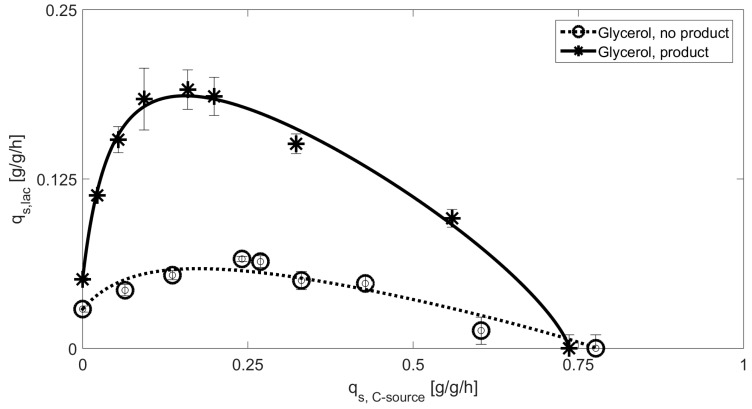
Extracted datapoints for q_s,C_ values including standard deviations for cultivations with glycerol using the product producing (glycerol, product) and the NP strain (glycerol, no product). Solid lines represent the model based approach for inducer uptake rates vs. feeding rates models of glucose.

The fermentations performed with the NP-strain showed lactose uptake rates resemble the expected carbon catabolite repression for glucose including high affinity of the PTS system at low q_s,glu_ which can also be seen in Table 3. Despite the identical behavior of protein producing and NP strain, a clear difference in maximum q_s,lac_ is obviously present. Higher consumption of glucose has impact on the cAMP level and decreases the specific uptake of lactose in the product producing strain. Y_X/S_ stays very similar in both cases 0.37 ± 0.05 for the protein producing strain vs. 0.383 ± 0.053 for the NP strain. Given yields are a mean value over all q_s,C_ values except for (lac) = 0 and (glu) = 0. So, these general deviations in q_s,lac,max_ can be attributed to the increased energy demand during recombinant product production, as also the biomass yields stay the same. Lactose uptake rates on glycerol for the product producing and the NP strain are given in Figure 4. Despite the quite straightforward mechanistic explanation for glucose, glycerol biomass to substrate yields differ fundamentally for both experiments: Y_X/S_ = 0.55 ± 0.11 for the NP strain, while the producing strain has a Y_X/S_ of 0.44 ± 0.1. This fact may explain the much shallower uptake at low q_s,C_ for the NP strain, but cannot explain the difference in the CCR term.

As a far higher biomass yield is present in the NP strain, only a reduced amount of lactose is taken up, which explains the decreased q_s,lac,max_. However, the NP strain shows no pronounced substrate inhibition. The carbon catabolite repression term of the model on glycerol has only low impact (see Table 3), as the upregulation of cAMP using glycerol would also be beneficial for the lactose uptake mechanism in the PTS system [35]. Since the lactose facilitator is not considered to be the rate determining step in the glycerol metabolism, glycerol kinase closely regulated to the PTS system may cause the CCR-like effects [44,45]. As the feeding rate increases, the possibility of short-term local glucose and glycerol accumulation increases, eventually leading to diauxic growth and therefore decreased lactose rates as glucose and glycerol have higher affinity than disaccharides for *E. coli* [35,52,57,58]. The product-producing strain shows a high regulated lactose uptake at low q_s,C_ values, as a result of lower biomass yield and higher energy demand in production of the recombinant protein. Higher lactose uptake results in high intracellular glucose level, which show the similar feedback mechanism like in the glucose fed cultivations.

As a result, both curves given in Figure 1 have a very similar appearance, but are expected to have a very different regulation within. To get insight into respiratory activity, qCO_2_ values are compared for all four fermentations, respectively. Evaluation is given in Table 4 based on the applied q_s,C_ values.

Highly similar respiratory activity is received for the product producing strain, almost linear increasing with q_s,C_. For the NP strain, a general lower respiratory activity is seen for the glycerol-fed strain. These results support the fact that lower energy demand is needed in this strain based on the general higher biomass yield and the fact that no recombinant protein is produced. In TCA, first steps of amino acid synthesis are performed, therefore the production of non-essential AA would result in the accumulation of NADH [59]. As approximately two NADH molecules can be formed to one molecule of CO_2_ the enhanced respiratory activity in the product producing strain is most likely coding for the enhanced production of non-essential AA, which are essential for the recombinant product. However, further analysis on stress induced changes in the gene expression may give valuable new insights into regulation mechanism in *E. coli*. 

### 3.2. Productivity and Physiology Using Glycerol as Primary Carbon Source

**Figure 5:**
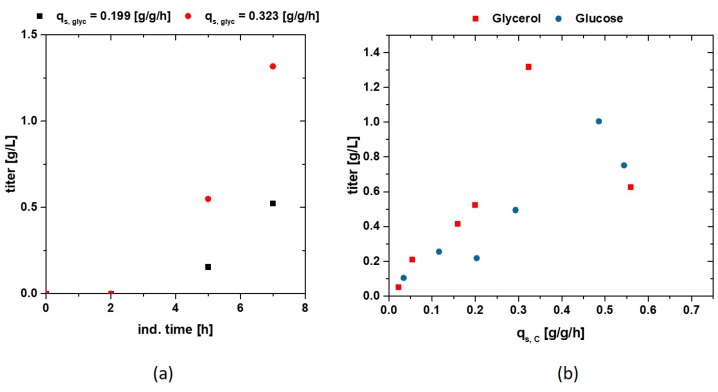
(**a**) Time dependence for two Inclusion Body (IB) titers starting from lactose addition to 7 h of induction; (**b**) Titers of the recombinant produced protein, after homogenisation of the inclusion bodies and a two-time washing plotted vs. the q_s_ of glucose and glycerol; A trend can be seen in gaining more product when cultivations are carried out on glycerol compared to glucose, respectively.

As the overall goal is an increased production rate of recombinant protein, we compare titers of the produced IBs as a function of carbon source and uptake rate. In Figure 5a, the increase in IB titer over time is presented for two cultivations. The loading of the induction, which takes about 2 h, can be clearly dedicated in these results, with no titer of the recombinant protein to be found within the first 2 h (also compare to Figure 2). Figure 5b shows product IB titers after 7 h induction time, which are plotted against the corresponding q_s,C_. Only the feed rate of glucose/glycerol, adapted for the static experiment in the induced fed-batch phase, is used in this plot—as cultivations are induced with one lactose pulse only, the q_s,C_ is a non-cumulative one. Generally, an increase in the feeding rate is beneficial for product formation. Cultivations carried out on glycerol tend to produce more recombinant protein with a product optimum at a q_s_-glycerol-level seen around 0.3–0.35 g/g/h. It may be possible that even higher product titers can be found within the range of 0.3–0.55 g/g/h. Cultivations carried out on glucose also tend to produce more product when the feeding rate is shifted to rather high rates as well. Very similar IB titers can be obtained at high q_s,C_ levels, but are far away from the observed maximum. 

The high increase in titer as a function of q_s,C_ in glycerol may be a result of the higher biomass (higher Y_X/S_ during induction) usually present in glycerol fed induction phases. The phenomenon of high product formation rates at high feeding levels, was much to our surprise, as we expected to see enhanced stress reactions by the cells due to overfeeding—especially at later time stages—usually present in IPTG induced cultures. Though we see only very little levels of glucose or glycerol accumulation in our HPLC measurements (data not shown). This could be, as the fermentation conditions in the induction phase are respectively mild. Temperature is decreased to 30 °C and induction with lactose is regarded to be a softer induction than IPTG, as lactose can be metabolized by *E. coli* [22,23]. In literature, it has been reported that the catabolic repression increases with higher temperatures [60]. Altering the temperature in the induction phase would have probably led to very different results in lactose uptake rates as well as different product data. Also, we want to highlight that every induction here was only performed with a one-time lactose pulse, which is most likely an insufficient induction, as there may be too little inducer in the media, which can be seen in Figure 2. In the following development steps, mixed feeds using glycerol in combination with lactose must be established and measured as this would lead to a constant and complete induction of the system. However, the product data supports the results that most probably very different regulation mechanisms in *E. coli* lead to the same visible uptake rates in Figure 1, but have severe effects on the productivity on the different carbon sources.

**Figure 6:**
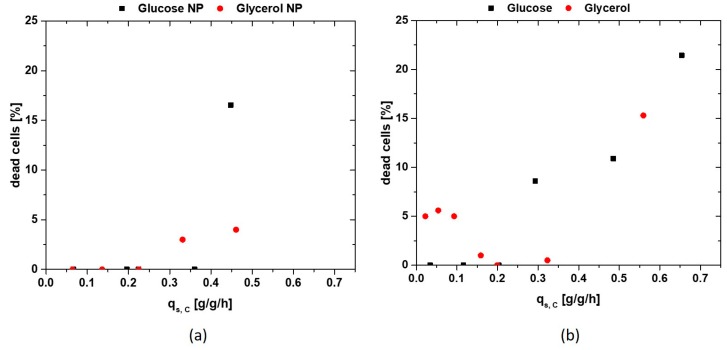
(**a**) Flow cytometry (FCM) analysis of NP strain 5 h after lactose pulse. As no protein data are received from these cultivations, the induction time was limited to 5 h; (**b**) FCM analysis of the product producing strain. Glycerol imposes stress at low feeding rates, while glucose shows increase in cell stress beginning at about 0.25 g/g/h.

Physiological analysis using flow cytometry (FCM) is presented in Figure 6a,b. The NP strain given in Figure 6a has very similar appearance for glucose and glycerol, respectively, increasing number of dead cells by increasing the feeding rate beyond a certain threshold, imposing stress to the cell. Throughout the whole experimental design, producing cells grown on glycerol exhibit a smaller cell size compared to cells grown on glucose (not shown). Since cell debris and residual particles are seen at similar cell sizes like glycerol grown cells a general higher abundance is present during those cultivations. To cope with this problem, FCM data after the non-induced fed-batch is subtracted from the subsequent measurements.

The viability of both cultivation strategies for an induction time of 6 h—often used for IB production at industrial scale—is given in Figure 6b, with a strong contrast between glucose and glycerol. While cells fed with glucose show no cell lysis at low q_s,C_ levels and are very similar to NP strain in Figure 6a, glycerol shows certain stress reaction resulting in about 5% dead cells until a 0.2 g/g/h. Afterwards, stable conditions for glycerol can be found, while stress is induced at glucose-fed systems starting at about 0.25 g/g/h. As the overnight fed-batch phase generally exhibited a q_s_ of 0.25 g/g/h, the switch to very low q_s,C_ in the induction phase, combined with the lactose pulse, may impose the cell stress seen in 5% dead cells in Figure 6b. This corresponds well to the product data in Figure 5 with similar or even higher productivity of glucose at low q_s,C_ levels, but higher productivity for glycerol at moderate to high levels. Including the fact that glycerol shows higher biomass yields during induction with lactose, glycerol may be well used as an alternative main carbon source in *E. coli* cultivations, even though glucose has high affinity to the phosphotransferase system (PTS). It has already been reported that addition of glycerol to a glucose-lactose induction system increases product formation [20,61]. As glycerol needs increased cAMP levels, which are also needed for lactose uptake [37], this might be a key function in regulating higher lactose uptake and subsequently increasing productivity and product titer. 

Furthermore, as glycerol is a cheap media compared to glucose, an application of glycerol in mixed-feed system with lactose may be highly beneficial for recombinant protein production performed in industry.

## 4. Conclusions

In this work, the effects of glycerol or glucose on lactose uptake rates for an IB-based process using *E. coli BL21*(*DE*3) were investigated. Feeding and uptake rates are compared and evaluated in terms of productivity and physiology using FCM. 

It is shown that both C-sources show identical lactose uptake rates as a function of q_s,C_. The used model-based approach already performed for different products in Wurm et al. [40] can be used for description of both curves. It has been detected that glycerol is beneficial over the usage of glucose for maximising the recombinant protein production of a lactose induced system. 

Glycerol and glucose most probably exhibit different regulation of the carbon catabolite repression—the reduction of lactose uptake at higher q_s,C_ levels. This hypothesis is supported by cultivation and evaluation of a non-producer strain exhibiting the expected behaviour for both C-sources, respectively. As this behaviour was not seen in the producing strain, it seems like the expression hosts are performing at maximum capacity in recombinant protein production. Additionally, glycerol is referred to different metabolic pathways [42], eventually increasing the metabolic flux [55] towards recombinant protein production.

Physiology and productivity support the hypothesis that glycerol is promising C-source for cultivations using mixed feed systems with moderate to high q_s,C_ values in order to boost time-space yields. As scale-up in *E. coli* systems can be performed relatively easily [1], the much lower costs of glycerol, when compared to glucose respectively, might provide interesting options for industrial and other large scale applications.

## Figures and Tables

**Table 1:** Respective sugar concentrations in media composition.

	Amount of Glucose	Amount of Glycerol
Preculture	8.8 g/L	8.9 g/L
Batch-Media	22 g/L	23 g/L
Feed	either 250 g/L or 300 g/L	

**Table 2:** Model parameters and normalized-root-mean-square-error (NRMSE) for the different analysed cultivation with produced product (P).

Cultivation System	q_s,lac,max_	K_A_	q_s,C,crit_	n	q_s,lac,noglu_	NRMSE
	[g/g/h]	[g/g/h]	[g/g/h]	[-]	[g/g/h]	[%]
Glucose	0.23	0.032	0.94	1.14	0.039	6.5
Glycerol	0.23	0.053	0.74	0.74	0.051	2.6

**Table 3:** Model parameters and normalized-root-mean-square-error (NRMSE) for the analysed cultivation without recombinant product production (NP).

Cultivation System	q_s,lac,max_ [g/g/h]	K_A_ [g/g/h]	q_s,glu,crit_ [g/g/h]	n [-]	q_s,lac,noglu_ [g/g/h]	NRMSE [%]
Glucose [NP]	0.14	0.016	0.96	2.92	0.032	12.7
Glycerol [NP]	0.10	0.13	0.78	0.90	0.029	9.7

**Table 4:** Specific substrate uptake rate vs. specific carbon evolution rate. Product producing strains have in general increased respiratory activity. NP strains show reduced respiratory activity. Standard deviation of qCO_2_ increases at higher feeding rates.

Glucose		Glucose NP		Glycerol		Glycerol NP	
q_s,C_ [g/g/h]	qCO_2_ [g/g/h]	q_s,C_ [g/g/h]	qCO_2_ [g/g/h]	q_s,C_ [g/g/h]	qCO_2_ [g/g/h]	q_s,C_ [g/g/h]	qCO_2_ [g/g/h]
0.036	2.15 ± 0.33	0.066	1.69 ± 0.25	0.022	2.91 ± 0.46	0.064	0.82 ± 0.09
0.116	3.12 ± 0.46	0.196	3.75 ± 0.44	0.054	4.41 ± 0.78	0.136	1.85 ± 0.21
0.197	3.98 ± 0.55	0.224	3.35 ± 0.42	0.093	3.88 ± 0.64	0.225	2.86 ± 0.31
0.286	5.72 ± 0.41	0.36	5.96 ± 0.26	0.159	3.12 ± 0.43	0.331	3.31 ± 0.22
0.403	6.42 ± 1.48	0.448	5.64 ± 0.47	0.199	4.14 ± 0.64	0.428	4.07 ± 0.51
0.544	7.30 ± 1.64			0.323	5.13 ± 0.48	0.603	1.75 ± 1.58
				0.559	7.18 ± 2.10		

## References

[1] Gupta S.K., Shukla P. (2017). Microbial platform technology for recombinant antibody fragment production: A review. Crit. Rev. Microbiol..

[2] Walsh G. (2010). Biopharmaceutical benchmarks 2010. Nat. Biotechnol..

[3] Wurm D.J., Veiter L., Ulonska S., Eggenreich B., Herwig C., Spadiut O. (2016). The *E. coli* pET expression system revisited-mechanistic correlation between glucose and lactose uptake. Appl. Microbiol. Biotechnol..

[4] Meuris L., Santens F., Elson G., Festjens N., Boone M., Dos Santos A., Devos S., Rousseau F., Plets E., Houthuys E. (2014). GlycoDelete engineering of mammalian cells simplifies N-glycosylation of recombinant proteins. Nat. Biotechnol..

[5] DeLisa M.P., Li J., Rao G., Weigand W.A., Bentley W.E. (1999). Monitoring GFP-operon fusion protein expression during high cell density cultivation of Escherichia coli using an on-line optical sensor. Biotechnol. Bioeng..

[6] Berlec A., Strukelj B. (2013). Current state and recent advances in biopharmaceutical production in *Escherichia coli*, yeasts and mammalian cells. J. Ind. Microbiol. Biotechnol..

[7] Baeshen M.N., Al-Hejin A.M., Bora R.S., Ahmed M.M., Ramadan H.A., Saini K.S., Baeshen N.A., Redwan E.M. (2015). Production of Biopharmaceuticals in *E. coli*: Current Scenario and Future Perspectives. J. Microbiol. Biotechnol..

[8] Spadiut O., Capone S., Krainer F., Glieder A., Herwig C. (2014). Microbials for the production of monoclonal antibodies and antibody fragments. Trends Biotechnol..

[9] Studier F.W., Moffatt B.A. (1986). Use of bacteriophage T7 RNA polymerase to direct selective high-level expression of cloned genes. J. Mol. Biol..

[10] Steen R., Dahlberg A.E., Lade B.N., Studier F.W., Dunn J.J. (1986). T7 RNA polymerase directed expression of the Escherichia coli rrnB operon. EMBO J..

[11] Studier F.W., Rosenberg A.H., Dunn J.J., Dubendorff J.W. (1990). Use of T7 RNA polymerase to direct expression of cloned genes. Methods Enzymol..

[12] Dubendorff J.W., Studier F.W. (1991). Controlling basal expression in an inducible T7 expression system by blocking the target T7 promoter with lac repressor. J. Mol. Biol..

[13] Neubauer P., Hofmann K. (1994). Efficient use of lactose for the lac promoter-controlled overexpression of the main antigenic protein of the foot and mouth disease virus in Escherichia coli under fed-batch fermentation conditions. FEMS Microbiol. Rev..

[14] Lyakhov D.L., He B., Zhang X., Studier F.W., Dunn J.J., McAllister W.T. (1998). Pausing and termination by bacteriophage T7 RNA polymerase. J. Mol. Biol..

[15] Jeong H., Barbe V., Lee C.H., Vallenet D., Yu D.S., Choi S.H., Couloux A., Lee S.W., Yoon S.H., Cattolico L. (2009). Genome sequences of *Escherichia coli* B strains REL606 and BL21(DE3). J. Mol. Biol..

[16] Jeong H., Kim H.J., Lee S.J. (2015). Complete Genome Sequence of *Escherichia coli* Strain BL21. Genome Announc..

[17] Tseng T.T., Tyler B.M., Setubal J.C. (2009). Protein secretion systems in bacterial-host associations, and their description in the Gene Ontology. BMC Microbiol..

[18] Marbach A., Bettenbrock K. (2012). Lac operon induction in *Escherichia coli*: Systematic comparison of IPTG and TMG induction and influence of the transacetylase LacA. J. Biotechnol..

[19] Keiler K.C. (2008). Biology of trans-translation. Annu. Rev. Microbiol..

[20] Viitanen M.I., Vasala A., Neubauer P., Alatossava T. (2003). Cheese whey-induced high-cell-density production of recombinant proteins in *Escherichia coli*. Microb. Cell Fact..

[21] Marisch K., Bayer K., Cserjan-Puschmann M., Luchner M., Striedner G. (2013). Evaluation of three industrial *Escherichia coli* strains in fed-batch cultivations during high-level SOD protein production. Microb. Cell Fact..

[22] Ukkonen K., Mayer S., Vasala A., Neubauer P. (2013). Use of slow glucose feeding as supporting carbon source in lactose autoinduction medium improves the robustness of protein expression at different aeration conditions. Protein Expr. Purif..

[23] Neubauer P., Hofmann K., Holst O., Mattiasson B., Kruschke P. (1992). Maximizing the expression of a recombinant gene in *Escherichia coli* by manipulation of induction time using lactose as inducer. Appl. Microbiol. Biotechnol..

[24] Marschall L., Sagmeister P., Herwig C. (2016). Tunable recombinant protein expression in *E. coli*: Enabler for continuous processing?. Appl. Microbiol. Biotechnol..

[25] Blommel P.G., Becker K.J., Duvnjak P., Fox B.G. (2007). Enhanced bacterial protein expression during auto-induction obtained by alteration of lac repressor dosage and medium composition. Biotechnol. Prog..

[26] Dvorak P., Chrast L., Nikel P.I., Fedr R., Soucek K., Sedlackova M., Chaloupkova R., de Lorenzo V., Prokop Z., Damborsky J. (2015). Exacerbation of substrate toxicity by IPTG in *Escherichia coli* BL21(DE3) carrying a synthetic metabolic pathway. Microb. Cell Fact..

[27] Wurm D.J., Herwig C., Spadiut O. (2017). How to Determine Interdependencies of Glucose and Lactose Uptake Rates for Heterologous Protein Production with *E. coli*. Methods Mol. Biol..

[28] García-Fruitós E., Vázquez E., Díez-Gil C., Corchero J.L., Seras-Franzoso J., Ratera I., Veciana J., Villaverde A. (2012). Bacterial inclusion bodies: Making gold from waste. Trends Biotechnol..

[29] Palmer I., Wingfield P.T. (2012). Preparation and extraction of insoluble (inclusion-body) proteins from *Escherichia coli*. Curr. Protoc. Protein Sci..

[30] Ramón A., Señorale-Pose M., Marín M. (2014). Inclusion bodies: Not that bad…. Front. Microbiol..

[31] Villaverde A., Corchero J.L., Seras-Franzoso J., Garcia-Fruitós E. (2015). Functional protein aggregates: Just the tip of the iceberg. Nanomedicine (Lond.).

[32] Wingfield P.T., Palmer I., Liang S.M. (2014). Folding and Purification of Insoluble (Inclusion Body) Proteins from *Escherichia coli*. Curr. Protoc. Protein Sci..

[33] Wingfield P.T. (2014). Preparation of Soluble Proteins from *Escherichia coli*. Curr. Protoc. Protein Sci..

[34] Postma P.W., Lengeler J.W., Jacobson G.R. (1993). Phosphoenolpyruvate:carbohydrate phosphotransferase systems of bacteria. Microbiol. Rev..

[35] Deutscher J., Francke C., Postma P.W. (2006). How phosphotransferase system-related protein phosphorylation regulates carbohydrate metabolism in bacteria. Microbiol. Mol. Biol. Rev..

[36] Ronimus R.S., Morgan H.W. (2003). Distribution and phylogenies of enzymes of the Embden-Meyerhof-Parnas pathway from archaea and hyperthermophilic bacteria support a gluconeogenic origin of metabolism. Archaea.

[37] Bettenbrock K., Fischer S., Kremling A., Jahreis K., Sauter T., Gilles E.D. (2006). A quantitative approach to catabolite repression in *Escherichia coli*. J. Biol. Chem..

[38] Kremling A., Bettenbrock K., Laube B., Jahreis K., Lengeler J.W., Gilles E.D. (2001). The organization of metabolic reaction networks. III. Application for diauxic growth on glucose and lactose. Metab. Eng..

[39] Stülke J., Hillen W. (1999). Carbon catabolite repression in bacteria. Curr. Opin. Microbiol..

[40] Wurm D.J., Hausjell J., Ulonska S., Herwig C., Spadiut O. (2017). Mechanistic platform knowledge of concomitant sugar uptake in *Escherichia coli* BL21(DE3) strains. Sci. Rep..

[41] Martínez-Gómez K., Flores N., Castañeda H.M., Martínez-Batallar G., Hernández-Chávez G., Ramírez O.T., Gosset G., Encarnación S., Bolivar F. (2012). New insights into *Escherichia coli* metabolism: Carbon scavenging, acetate metabolism and carbon recycling responses during growth on glycerol. Microb. Cell Fact..

[42] Lin E.C. (1976). Glycerol dissimilation and its regulation in bacteria. Annu. Rev. Microbiol..

[43] Achmüller C., Kaar W., Ahrer K., Wechner P., Hahn R., Werther F., Schmidinger H., Cserjan-Puschmann M., Clementschitsch F., Striedner G. (2007). N(pro) fusion technology to produce proteins with authentic N termini in *E. coli*. Nat. Methods.

[44] Zwaig N., Kistler W.S., Lin E.C. (1970). Glycerol kinase, the pacemaker for the dissimilation of glycerol in *Escherichia coli*. J. Bacteriol..

[45] Voegele R.T., Sweet G.D., Boos W. (1993). Glycerol kinase of *Escherichia coli* is activated by interaction with the glycerol facilitator. J. Bacteriol..

[46] Slouka C., Wurm D.J., Brunauer G., Welzl-Wachter A., Spadiut O., Fleig J., Herwig C. (2016). A Novel Application for Low Frequency Electrochemical Impedance Spectroscopy as an Online Process Monitoring Tool for Viable Cell Concentrations. Sensors (Basel).

[47] Langemann T., Mayr U.B., Meitz A., Lubitz W., Herwig C. (2016). Multi-parameter flow cytometry as a process analytical technology (PAT) approach for the assessment of bacterial ghost production. Appl. Microbiol. Biotechnol..

[48] Xu J., Banerjee A., Pan S.H., Li Z.J. (2012). Galactose can be an inducer for production of therapeutic proteins by auto-induction using *E. coli* BL21 strains. Protein Expr. Purif..

[49] Studier F.W., Daegelen P., Lenski R.E., Maslov S., Kim J.F. (2009). Understanding the differences between genome sequences of *Escherichia coli* B strains REL606 and BL21(DE3) and comparison of the *E. coli* B and K-12 genomes. J. Mol. Biol..

[50] Daegelen P., Studier F.W., Lenski R.E., Cure S., Kim J.F. (2009). Tracing ancestors and relatives of *Escherichia coli* B, and the derivation of B strains REL606 and BL21(DE3). J. Mol. Biol..

[51] Korz D.J., Rinas U., Hellmuth K., Sanders E.A., Deckwer W.D. (1995). Simple fed-batch technique for high cell density cultivation of *Escherichia coli*. J. Biotechnol..

[52] Inada T., Kimata K., Aiba H. (1996). Mechanism responsible for glucose-lactose diauxie in *Escherichia coli*: Challenge to the cAMP model. Genes Cells.

[53] Larson T.J., Ye S.Z., Weissenborn D.L., Hoffmann H.J., Schweizer H. (1987). Purification and characterization of the repressor for the sn-glycerol 3-phosphate regulon of *Escherichia coli* K12. J. Biol. Chem..

[54] Iuchi S., Cole S.T., Lin E.C. (1990). Multiple regulatory elements for the glpA operon encoding anaerobic glycerol-3-phosphate dehydrogenase and the glpD operon encoding aerobic glycerol-3-phosphate dehydrogenase in *Escherichia coli*: Further characterization of respiratory control. J. Bacteriol..

[55] Heyland J., Blank L.M., Schmid A. (2011). Quantification of metabolic limitations during recombinant protein production in *Escherichia coli*. J. Biotechnol..

[56] Glick B.R. (1995). Metabolic load and heterologous gene expression. Biotechnol. Adv..

[57] Weissenborn D.L., Wittekindt N., Larson T.J. (1992). Structure and regulation of the glpFK operon encoding glycerol diffusion facilitator and glycerol kinase of *Escherichia coli* K-12. J. Biol. Chem..

[58] Hogema B.M., Arents J.C., Bader R., Postma P.W. (1999). Autoregulation of lactose uptake through the LacY permease by enzyme IIAGlc of the PTS in *Escherichia coli* K-12. Mol. Microbiol..

[59] Berg J.M., Tymoczko J.L., Stryer L. (2002). Biochemistry.

[60] Marr A.G., Ingraham J.L., Squires C.L. (1964). Effect of the temperature of growth of *Escherichia coli* on the formation of beta-galactosidase. J. Bacteriol..

[61] Mayer S., Junne S., Ukkonen K., Glazyrina J., Glauche F., Neubauer P., Vasala A. (2014). Lactose autoinduction with enzymatic glucose release: Characterization of the cultivation system in bioreactor. Protein Expr. Purif..

